# An Magnetic Resonance Imaging–directed Targeted-plus-perilesional Biopsy Approach for Prostate Cancer Diagnosis: “Less Is More”

**DOI:** 10.1016/j.euros.2022.07.006

**Published:** 2022-08-02

**Authors:** Marinus J. Hagens, M. Arjen Noordzij, Jan Willem Mazel, Auke Jager, Thierry N. Boellaard, Jeroen A.W. Tielbeek, Margot Henebiens, Ivo G. Schoots, Pim J. van Leeuwen, Henk G. van der Poel, Sybren P. Rynja

**Affiliations:** a Department of Urology, Amsterdam UMC Location Vrije Universiteit Amsterdam, Amsterdam, The Netherlands; b Department of Urology, Netherlands Cancer Institute - Antoni van Leeuwenhoek Hospital (NCI-AVL), Amsterdam, The Netherlands; c Prostate Cancer Network Netherlands, Amsterdam, The Netherlands; d Department of Urology, Spaarne Gasthuis, Haarlem and Hoofddorp, The Netherlands; e Department of Radiology, Netherlands Cancer Institute – Antoni van Leeuwenhoek Hospital (NCI-AVL), Amsterdam, The Netherlands; f Department of Radiology, Spaarne Gasthuis, Haarlem and Hoofddorp, The Netherlands; g Department of Radiology and Nuclear Medicine, Erasmus University Medical Center, Rotterdam, The Netherlands

**Keywords:** Prostate biopsies, Transperineal, Cognitive fusion, Perilesional systematic biopsies

## Abstract

**Background:**

Considering that most men benefit diagnostically from increased sampling of index lesions, limiting systematic biopsy (SBx) to the region around the index lesion could potentially minimize overdetection while maintaining the detection of clinically significant prostate cancer (csPCa).

**Objective:**

To evaluate the diagnostic performance of a hypothetical magnetic resonance imaging (MRI)-directed targeted-plus-perilesional biopsy approach.

**Design, setting, and participants:**

This single-center, retrospective analysis of prospectively generated data included all biopsy-naïve men with unilateral MRI-positive lesions (Prostate Imaging Reporting and Data System category ≥3), undergoing both MRI-directed targeted biopsies and SBx. Grade group 2–5 cancers were considered csPCa.

**Outcome measurements and statistical analysis:**

The diagnostic performance of a targeted-plus-perilesional biopsy approach was compared with that of a targeted-plus-systematic biopsy approach. The primary outcome was the detection of csPCa. Secondary outcomes included the detection of clinically insignificant prostate cancer (ciPCa) and the number of total biopsy cores.

**Results and limitations:**

A total of 235 men were included in the analysis; csPCa and ciPCa were detected, respectively, in 95 (40.4%) and 86 (36.6%) of these 235 men. A targeted-plus-perilesional biopsy approach would have detected 92/95 (96.8%; 95% confidence interval [CI] 91.0–99.3%) csPCa cases. At the same time, detection of systematically found ciPCa would be reduced by 11/86 (12.8%; 95% CI 6.6–21.7%). If a targeted-plus-perilesional biopsy approach would have been performed, the number of biopsy cores per patient would have been reduced significantly (a mean difference of 5.2; 95% CI 4.9–5.6, *p* < 0.001).

**Conclusions:**

An MRI-directed targeted-plus-perilesional biopsy approach detected almost all csPCa cases, while limiting overdiagnosis and reducing the number of biopsy cores. Prospective clinical trials are needed to substantiate the withholding of nonperilesional SBx in men with unilateral lesion(s) on MRI.

**Patient summary:**

Limiting systematic biopsies to the proximity of the suspicious area on magnetic resonance imaging helps detect an equivalent number of aggressive cancers and fewer indolent cancers. These findings may help patients and physicians choose the best biopsy approach.

## Take Home Message

This retrospective study found arguments to limit systematic biopsies only to ipsilateral prostate lobe sectors directly adjacent to the magnetic resonance imaging (MRI)-positive lesion. An MRI-directed targeted-plus-perilesional biopsy approach detects clinically significant prostate cancer cases equivalent to the recommended practice of an MRI-directed targeted-plus-systematic biopsy approach, while reducing overdiagnosis rates and the number of biopsy cores.

## 1 Introduction

Magnetic resonance imaging (MRI) of the prostate is increasingly being used in men suspected of having prostate cancer (PCa) to target prostate biopsies, either by cognitive or software-assisted fusion approaches [1], [2]. Even though recent studies have shown that the use of targeted biopsy (TBx) increases the detection of clinically significant PCa (csPCa), still a substantial proportion of csPCa is missed by TBx alone [3], [4]. It remains unknown whether csPCa is missed due to the limited sensitivity of MRI, the suboptimal image fusion, the biopsy technique and strategy, or a combination of these. Therefore, complementary systematic biopsy (SBx) is still recommended by the European Association of Urology (EAU) PCa guidelines to maximize the detection of csPCa [1].

Complementary SBx increases the detection of csPCa; however, it also increases the detection of clinically insignificant PCa (ciPCa) [4], [5], [6]. Given its very low metastatic potential and consequently very low risk of cancer-specific mortality, detection of ciPCa is generally considered overdiagnosis, and is associated with overtreatment and a high patient burden [7]. Furthermore, the increased number of biopsy cores, when performing both TBx and SBx, is associated with a longer procedure time, more pronounced patient discomfort, increased workload for pathologists, and increased costs [8], [9], [10]. In view of the abovementioned disadvantages, the traditional whole-gland SBx template is currently being disputed [6], [11], [12].

Ideally, prostate biopsy strategies maximize the detection of csPCa, while minimizing the detection of ciPCa. Considering that most men benefit diagnostically from increased sampling of index lesions, limiting SBx to the region around the index lesion could potentially effectuate this [13], [14]. This study aims to retrospectively evaluate the diagnostic performance of TBx + perilesional SBx, compared with TBx + standard SBx, in men with MRI-positive unilateral lesion(s) suggestive of being csPCa.

## 2 Patients and methods

### 2.1 Study design and population

This retrospective cohort study was approved by the institutional review board (IRBd21-031), and the requirement to obtain informed consent was waived. Consecutive men, suspected of having PCa, were enrolled in a prospective database assessing the accuracy of cognitive transperineal MRI-directed prostate biopsies at the teaching hospital Spaarne Gasthuis Hoofddorp between February 2020 and February 2022. The present study included only biopsy-naïve men with MRI-positive unilateral lesion(s) in whom both TBx and SBx were performed. MRI positivity was defined as Prostate Imaging Reporting and Data System (PI-RADS) assessment category ≥3. Men in whom SBx cores were taken only from the ipsilateral side of the prostate, relative to the MRI-positive lesion, were excluded from the analysis.

### 2.2 Image acquisition and analysis

Prior to prostate biopsies, all men underwent MRI using a 3 Tesla Siemens Magnetom Verio or a 1.5 Tesla Philips Ambition in the event that a 3 Tesla was contraindicated. The European Society of Urogenital Radiology MRI protocol was used containing axial fast spin-echo T1-weighted images of the pelvis [15]. T2-weighted fast recovery turbo spin-echo images of the prostate were acquired in the axial, sagittal, and coronal planes (slice thickness 3 mm). Axial diffusion-weighted imaging was performed using a spin-echo echo-planar imaging pulse sequence with slice thickness 5 mm (*b* values: *b*-0, *b*-1000 or *b*-1400, *b*-2000 s/mm^2^); apparent diffusion coefficient maps were calculated automatically.

Images were prospectively interpreted by seven experienced radiologists, all of whom have >3 yr of experience in reading prostate MRI. The standardized five-point PI-RADS classification was used, in concordance with the PI-RADS version 2.1 guidelines [16]. If multiple unilateral MRI-positive lesions were observed, the lesion with the highest PI-RADS assessment category was designated as the index lesion and used for analysis. If multiple MRI-positive lesions had the highest PI-RADS assessment category, the largest lesion was designated as the index lesion and used for analysis.

### 2.3 MRI/ultrasound-fusion biopsy and histopathology

Transperineal prostate biopsy procedures were performed using a free-hand cognitive fusion approach based on the Ginsburg protocol [17]. Biopsies were taken under local anesthesia using a spring-loaded biopsy gun with an 18-G needle. Antibiotic prophylaxis was administered only if infectious features were found in urinalysis (urine sediment and/or urine culture).

**Fig. 1:**
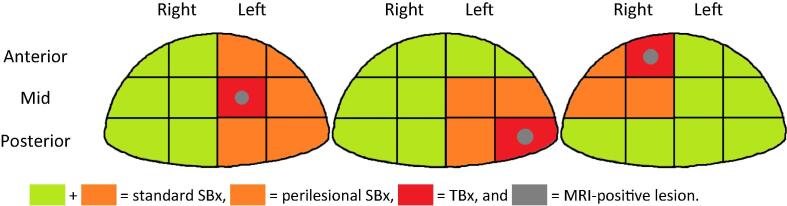
Graphical representation of TBx, TBx + standard SBx, and TBx + perilesional SBx. MRI = magnetic resonance imaging; SBx systematic biopsies; TBx = targeted biopsies.

A median of five TBx cores were taken from the MRI-positive index lesion. During the same session, a median of eight SBx cores were obtained from the MRI-negative regions on both prostate lobes. All procedures were performed by one of three experienced urologists with ≥2 yr of experience in transperineal cognitive fusion biopsies. The urologist who performed the biopsies had access to all MRI data and visually mapped the lesion of interest in the Ginsburg protocol. In addition to the reference of TBx + standard SBx, a hypothetical biopsy template was defined: TBx + perilesional SBx (Fig. 1 and Supplementary Fig. 1). Based on pre-existing literature, perilesional SBx cores were defined as all nontargeted biopsy cores taken from the ipsilateral prostate lobe sectors directly adjacent to the MRI-positive index lesion [13].

Core-needle biopsy specimens were potted and reported separately according to the sector these originated from. All specimens were prospectively analyzed by experienced uropathologists and reported according to the International Society of Urological Pathology (ISUP) consensus for grading of PCa [18]. Clinically significant PCa was defined as Gleason score ≥3 + 4, equivalent to ISUP grade group (GG) ≥2. Additionally, a second stricter definition of csPCa was used, equivalent to ISUP GG ≥3.

### 2.4 Statistical analysis

Categorical variables were reported as frequency distributions and percentages, and continuous variables were expressed as medians with interquartile ranges (IQRs).

Detection rates of ciPCa and csPCa were calculated for both TBx and TBx + perilesional SBx approaches, and compared with the detection on TBx + standard SBx as the “gold standard.” Sensitivities and 95% confidence intervals (CIs) of detection rates were calculated using binomial tests, and intergroup differences between sensitivities were considered statistically significant if the 95% CIs did not overlap. A subgroup analysis was performed for the PI-RADS assessment category. Differences between subgroup detection rates were compared with the Fisher’s exact test using the Freeman-Halton extension. All statistical analyses were performed with the statistical package SPSS for MacOS, version 27 (IBM, Armonk, NY, USA).

## 3 Results

A total of 235 men were retrospectively included for analysis. Patient characteristics are presented in Table 1.

**Table 1:** Patient characteristics (*n* = 235)

Age (yr)		69 (63–73)
DRE positive (*n*)		91 (38.7)
Initial PSA (ng/ml)		7.8 (5.6–14.0)
Prostate volume (ml)		45.0 (34.0–65.8)
PSA density (ng/ml/ml)		0.17(0.11–0.25)
Number of TBx + SBx per patient (*n*)		12 (10–15)
Number of TBx per patient (*n*)		5 (4–6)
Number of SBx per patient (*n*)		8 (5–10)
Number of TBx + PLBx per patient (*n*)		7 (6–9)
PI-RADS category	3	33 (14.0)
	4	119 (50.6)
	5	83 (35.3)
ISUP grade group	Benign	54 (23.0)
	1	86 (36.6)
	2	48 (20.4)
	3	21 (8.9)
	4	16 (6.8)
	5	10 (4.3)

DRE = digital rectal examination; IQR = interquartile range; ISUP = International Society of Urological Pathology; PI-RADS = Prostate Imaging Reporting and Data System; PLBx = perilesional biopsies; PSA = prostate-specific antigen; SBx = systematic biopsies; TBx = targeted biopsies.

All continuous variables are reported as median (IQR) and all categorical variables are reported as *n* (%).

### 3.1 Diagnostic performance TBx + perilesional SBx

Using the current “reference standard” of TBx + standard SBx, among 235 men, csPCa was detected in 95 (40.4%), ciPCa was detected in 86 (36.6%), and no PCa was detected in 54 (23.0%). These cancer detection rates were achieved using a median of 5 (IQR 4–6) TBx cores and a median of 8 (IQR 5–10) SBx cores, adding up to a median of 12 (IQR 10–15) total biopsy cores per patient.

Compared with a TBx + standard SBx approach, a TBx + perilesional SBx approach would have correctly detected 92/95 (96.8%; 95% CI 91.0–99.3%) csPCa cases (Table 2). Performing only additional perilesional SBx, csPCa would have been overlooked most frequently in men with PI-RADS 3 lesions (16.7%; 95% CI 0.4–64.1%), compared with men with PI-RADS 4 (2.5%; 95% CI 0.1–13.2%) and PI-RADS 5 (2.0%; 95% CI 0.1–10.9%) lesions (Table 3). In the case of discrepancy in the detection of csPCa between TBx + standard SBx and TBx + perilesional SBx approaches, the highest ISUP GG was found in the contralateral sectors directly adjacent to the MRI-positive lesion (ie, if the MRI-positive lesion is located in the right posterior sector, the highest ISUP GG is found in the contralateral adjacent posterior sector; Supplementary Table 1). Moreover, all the detected nonperilesional csPCa cases involved ISUP GG 2 cancers. Consequently, if a stricter definition of csPCa was used, equivalent to ISUP GG ≥3, a TBx + perilesional SBx approach would have correctly detected 47/47 (100.0%; 95% CI 92.5–100.0%) csPCa cases (Supplementary Table 2).

**Table 2:** Cancer detection rates of TBx + perilesional SBx in reference to TBx + standard SBx

		TBx + standard SBx			Total
		csPCa (GG ≥2)	ciPCa (GG = 1)	Benign	
TBx + perilesional SBx	csPCa (GG ≥2)	92	0	0	92
	ciPCa (GG = 1)	*3*	75	0	78
	Benign	0	*11*	54	65
Total		95	86	54	235

ciPCa = clinically insignificant prostate cancer; csPCa = clinically significant prostate cancer; GG = grade group; SBx = systematic biopsies; TBx = targeted biopsies.

**Table 3:** Cancer detection rates of TBx + perilesional SBx by PI-RADS category in reference to TBx + standard SBx

			TBx + standard SBx			Total
			csPCa (GG ≥2)	ciPCa (GG = 1)	Benign	
PI-RADS 3	TBx + perilesional SBx	csPCa (GG ≥2)	5	0	0	5
		ciPCa (GG = 1)	*1*	6	0	7
		Benign	0	*3*	18	21
	Total		6	9	18	33
PI-RADS 4	TBx + perilesional SBx	csPCa (GG ≥2)	39	0	0	39
		ciPCa (GG = 1)	*1*	45	0	46
		Benign	0	*7*	27	34
	Total		40	52	27	119
PI-RADS 5	TBx + perilesional SBx	csPCa (GG ≥2)	48	0	0	48
		ciPCa (GG = 1)	*1*	24	0	25
		Benign	0	*1*	9	10
	Total		49	25	9	83

ciPCa = clinically insignificant prostate cancer; csPCa = clinically significant prostate cancer; GG = grade group; PI-RADS = Prostate Imaging Reporting and Data System; SBx = systematic biopsies; TBx = targeted biopsies.

Compared with TBx, standard and perilesional SBx minimally increased the csPCa detection rate, as six of 95 (6.3%) csPCa diagnoses were found systematically (Supplementary Table 3). Particularly in men with PI-RADS 4 and 5 lesions, standard and perilesional SBx were of limited or no added value. In contrast, standard and perilesional SBx were of greater added value in men with PI-RADS 3 lesions (Supplementary Table 4).

Using a TBx + perilesional SBx approach, detection of ciPCa would have been reduced substantially. Limiting SBx to ipsilateral prostate lobe sectors directly adjacent to the MRI-positive lesion would have reduced the systematically found ciPCa from 14 to three (difference 12.8%; 95% CI 6.6–21.7%) of 86 men. Nonperilesional SBx detected small ciPCa lesions in these 11 cases, as in most cases only one or two biopsy cores showed positive findings.

### 3.2 Number of biopsy cores

When performing only perilesional SBx in addition to TBx, the total number of biopsy cores per patient decreased by 38.5% (IQR 30.0–50.0%); a median number of 7 (IQR 6–9) biopsy cores were taken using the TBx + perilesional SBx approach. Compared with the TBx + standard SBx approach, this resulted in a significant reduction of 5.2 (*p* < 0.001; 95% CI 4.9–5.6) biopsy cores per patient on average.

## 4 Discussion

Our findings suggest that a TBx + perilesional SBx approach would be a good alternative that reduces the detection of ciPCa and the total number of biopsy cores, while preserving the detection of csPCa. Moreover, based on the data presented, a further reduction in the number of biopsies could be reached in men with PI-RADS 4 and 5 lesions.

To maximize the detection of csPCa, standard SBx as a supplement to TBx is currently still recommended by EAU guidelines on PCa diagnosis [1], [11], [12], [19]. The addition of SBx, however, not only improves the detection of csPCa, but also coincides with an increased detection of ciPCa [20], [21]. Possible explanations for missing csPCa on TBx are related to imprecise lesion registration (underestimation of tumor volume) and targeting errors due to cognitive fusion inaccuracies [19], [21], [22]. Considering that most of the csPCa missed by TBx are found in sextants adjacent to MRI-positive lesions, limiting SBx to the vicinity of MRI-positive lesions has been suggested as an alternative to standard “whole-prostate” SBx.

Given the lack of consensus on how to implement a TBx + perilesional SBx approach, different interpretations exist in the current literature, differing in both the suggested numbers and the locations of perilesional SBx [13]. Owing to the high level of skill and expertise required for previously proposed templates, applicability has been limited so far. This study’s proposed TBx + perilesional SBx approach used equivalent numbers of biopsy cores to those reported in previously published studies [6], [12], [23], but differed in the proposed locations. Limiting SBx to the ipsilateral prostate lobe sectors directly adjacent to the MRI-positive lesion, which requires less skill and expertise, will improve the applicability and usefulness compared with previously proposed approaches.

The csPCa detection rate of TBx within this cohort is notable. By performing only TBx and thus omitting SBx altogether, 89/95 (93.7%; 95% CI 86.8–97.6%) cases of csPCa would have been classified correctly. This is substantially higher than that reported in the existing literature, where detection rates of csPCa for TBx range between 67% and 89% [13]. The relatively high number of TBx (five biopsy cores; IQR four to six) taken per patient within this cohort, making it comparable with an extended TBx or focal saturation approach, could be the explanation [11]. Increasing the number of TBx events also overcomes the problem of imprecise lesion registration and targeting errors, rendering complementary perilesional SBx redundant. However, good image quality is of paramount importance. Poor image quality greatly complicates the registration and targeting of lesions. Ideally, before implementing a TBx (+perilesional SBx) approach, image quality should be ensured by using a scoring system (Prostate Imaging Quality [PI-QUAL] score or in-house scoring system) [24]. This study did not use a scoring system but rather a binomial determination of the image quality: insufficient (no PI-RADS score) or sufficient (a PI-RADS score).

Preventing overdiagnosis of ciPCa also prevents overtreatment and the associated impaired quality of life due to urinary, sexual, or gastrointestinal side effects. Limiting the number of SBx events substantially reduces overdiagnosis rates; 12.8% of men in our present study would have avoided a diagnosis of ciPCa. Additionally, reducing the number of biopsy cores would also reduce workload for pathologists, shorten procedure time, and minimize patient discomfort [9], [10]. Considering the fact that perilesional SBx had limited or no added value in PI-RADS 4 and 5 lesions within this cohort, it could be argued that the number of biopsies can be reduced further in these subgroups. By performing only perilesional SBx in men with PI-RADS 3 lesions and limiting prostate biopsies in men with PI-RADS 4 and 5 lesions to TBx only, the reduction in the total number of biopsies per patient could be increased further to 61.5% (IQR 50.0–70.0%). However, because a hypothetical biopsy template was used in this study, we were unable to provide the necessary data to substantiate these arguments.

Our study is not devoid of limitations. To start, since we used a prospective database in which transperineal prostate biopsies were reviewed, definitive histopathological verification from prostatectomy specimens was lacking. Although the Ginsburg protocol reliably detects 97% of csPCa later found at prostatectomy [25], the lack of definitive histopathology limits the findings of this study. Owing to daily practice, MRI scans were performed on two different scanners and interpreted by different radiologists, which may have influenced the findings. The accuracy of the MRI reading may vary with changes in technical equipment and by the interpretation of different observers [26]. All prostate MRI scans were read by seven experienced radiologists, although the experience in reading prostate MRI varied among the radiologists. All seven radiologists have read sufficient MRI scans to be experienced enough to be an independent reader, and six out of seven have read at least 300 MRI scans [27]. Interpretation by experienced but nonexpert readers may have influenced the findings. This cohort used biparametric MRI acquisition rather than multiparametric MRI acquisition. The lack of dynamic contrast enhanced (DCE) sequences may have affected MRI interpretation; in the presence of a DCE sequence, a PI-RADS 3 lesion might have been interpreted as a PI-RADS 4 lesion. In addition, the proposed TBx + perilesional SBx approach relies on the presence of an MRI-positive lesion, which limits its applicability; men with negative MRI, but still a high clinical suspicion, need different biopsy approaches. Another limitation that merits discussion is the therapeutic impact of such a TBx + perilesional SBx approach. It is unknown whether it is better to reduce the number of biopsy cores or improve prostate mapping, to perform a more confident and safer treatment. Finally, given the retrospective nature of this study, results need to be interpreted with caution. Future prospective cohort studies are therefore needed to further investigate and optimize this promising diagnostic biopsy approach.

## 5 Conclusions

Limiting systematic biopsies only to ipsilateral prostate lobe sectors directly adjacent to the MRI-positive lesion, thereby avoiding contralateral systematic biopsies altogether, has shown to be a promising diagnostic biopsy approach in biopsy-naïve men suspected of having csPCa. Increased sampling of the index lesion correctly detects almost all csPCa cases, while limiting the overdiagnosis of ciPCa. Future studies, using prostatectomy specimens as histopathological verification, focusing on its applicability and therapeutic impact are needed to facilitate the adoption of this proposed biopsy approach.

  ***Author contributions*:** Marinus J. Hagens had full access to all the data in the study and takes responsibility for the integrity of the data and the accuracy of the data analysis.

*Study concept and design*: Hagens, Schoots, Rynja, van der Poel, van Leeuwen.

*Acquisition of data*: Hagens, Rynja, Noordzij, Mazel.

*Analysis and interpretation of data*: Hagens, Rynja, van Leeuwen, van der Poel.

*Drafting of the manuscript*: Hagens, Rynja, van Leeuwen, van der Poel.

*Critical revision of the manuscript for important intellectual content*: Schoots, van der Poel, van Leeuwen, Noordzij, Mazel, Jager, Boellaard, Tielbeek, Henebiens, Rynja.

*Statistical analysis*: Hagens.

*Obtaining funding*: None.

*Administrative, technical, or material support*: None.

*Supervision*: Rynja, van Leeuwen, van der Poel.

*Other*: None.

  ***Financial disclosures:*** Marinus J. Hagens certifies that all conflicts of interest, including specific financial interests and relationships and affiliations relevant to the subject matter or materials discussed in the manuscript (eg, employment/affiliation, grants or funding, consultancies, honoraria, stock ownership or options, expert testimony, royalties, or patents filed, received, or pending), are the following: None.

  ***Funding/Support and role of the sponsor:*** None.
