# Supplementary material for: An Magnetic Resonance Imaging–directed Targeted-plus-perilesional Biopsy Approach for Prostate Cancer Diagnosis: “Less Is More”
Source: Eur Urol Open Sci. 2022 Aug 2;43:68–73. doi: 10.1016/j.euros.2022.07.006 (PMC9638771; doi:10.1016/j.euros.2022.07.006)
Supplement: Supplementary Fig. 1 [file mmc1.docx]

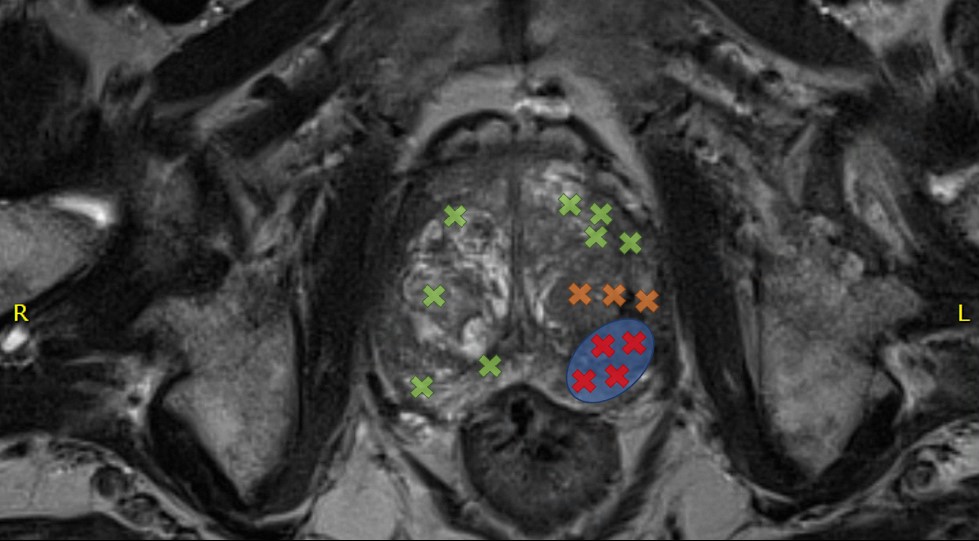


**Supplementary Figure 1A.** Illustrative representation of an existing case with a PI-RADS 5 lesion left posteriorly (indicated in blue). All TBx (indicated as red crosses) yielded benign prostate tissue, while all perilesional SBx (indicated as orange crosses) yielded ISUP GG 4 PCa. Standard SBx (indicated as green crosses) also yielded benign prostate tissue. In hindsight, it is believed that TBx had been targeted too posteriorly (targeting error), but with the use of perilesional SBx the lesion could still be correctly identified.


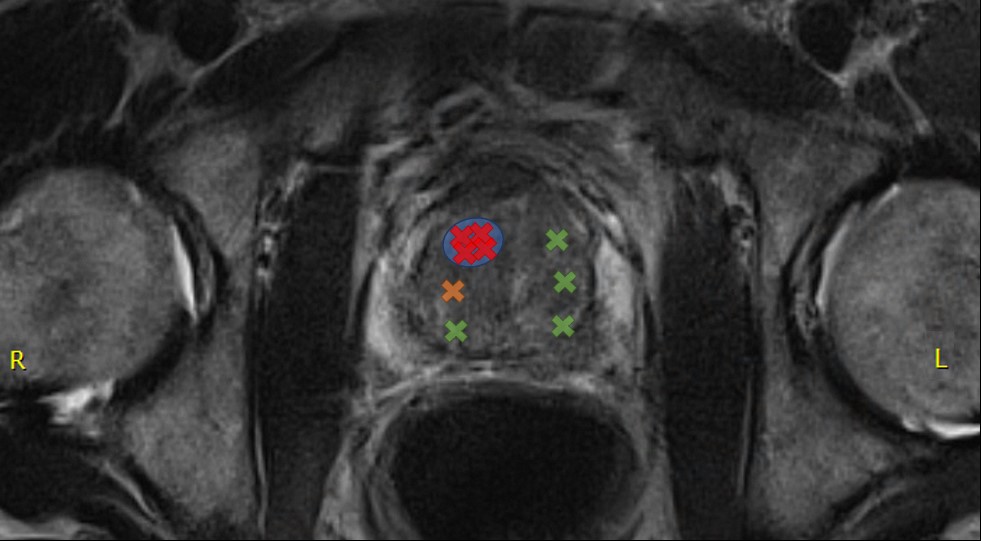


**Supplementary Figure 1B.** Illustrative representation of an existing case with a PI-RADS 4 lesions right anteriorly (indicated in blue). All TBx and perilesional SBx (indicated as red and orange crosses, respectively) yielded benign prostate tissue, while one standard SBx (indicated as green crosses) yielded ISUP GG 1 PCa; the left posterior SBx.


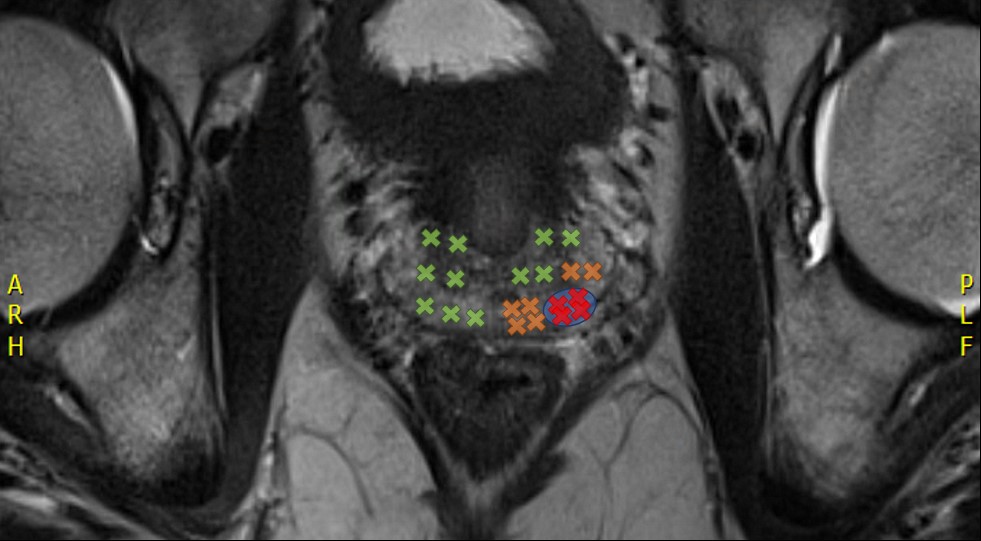


**Supplementary Figure 1C**. Illustrative representation of an existing case with a PI-RADS 4 lesion left posteriorly (indicated in blue). All TBx and perilesional SBx (indicated as red and orange crosses, respectively) yielded benign prostate tissue, while two standard SBx (indicated as green crosses) yielded ISUP GG 1 PCa; one right posterior SBx and one left anterior SBx.
