# Supplementary material for: An Magnetic Resonance Imaging–directed Targeted-plus-perilesional Biopsy Approach for Prostate Cancer Diagnosis: “Less Is More”
Source: Eur Urol Open Sci. 2022 Aug 2;43:68–73. doi: 10.1016/j.euros.2022.07.006 (PMC9638771; doi:10.1016/j.euros.2022.07.006)
Supplement: Supplementary Tables [file mmc2.docx]

| Supplementary Table 1. Characteristics and descriptions of csPCa missed by TBx + perilesional SBx approach | | | | | |
| --- | --- | --- | --- | --- | --- |
|  | iPSA | PSAD | TBx + perilesional SBx outcome | TBx + standard SBx outcome | Description |
| **1** | 18.3 | 0.68 | 3+3=6, ISUP 1 | 3+4=7, ISUP 2 | PI-RADS 3 lesion in the left posterior sector with ISUP 1 cancer detected with TBx + perilesional SBx. ISUP 2 cancer was detected with standard SBx in the contralateral adjacent posterior sector. |
| **2** | 3.1 | 0.10 | 3+3=6, ISUP 1 | 3+4=7, ISUP 2 | PI-RADS 4 lesion in the right posterior sector with ISUP 1 cancer detected with TBx + perilesional SBx. ISUP 2 cancer was detected with standard SBx in the contralateral adjacent posterior sector. |
| **3** | 68.5 | 2.36 | 3+3=6, ISUP 1 | 3+4=7, ISUP 2 | PI-RADS 5 lesion in the right anterior and mid sectors with ISUP 1 cancer detected with TBx + perilesional SBx. ISUP 2 cancer was detected with standard SBx in the contralateral adjacent mid sector. |

**Supplementary Table 1.** Characteristics and descriptions of csPCa missed by TBx + perilesional SBx approach. iPSA initial prostate specific antigen, PSAD prostate specific antigen density, TBx targeted biopsies, SBx systematic biopsies, ISUP international society of urological pathology, PI-RADS prostate imaging reporting and data system.

| Supplementary Table 2. Cancer detection rates using ISUP GG ≥3 as threshold for csPCa | | | | | |
| --- | --- | --- | --- | --- | --- |
|  | | **TBx + Standard SBx** | | |  |
|  |  | csPCa (GG≥2) | ciPCa (GG=1) | Benign | Total |
| **TBx + Perilesional SBx** | csPCa (GG≥3) | 47 | 0 | 0 | 47 |
|  | ciPCa (GG≤2) | *0* | 123 | 0 | 123 |
|  | Benign | 0 | *11* | 54 | 65 |
| **Total** | | 47 | 134 | 54 | 235 |

**Supplementary Table 2.** Cancer detection rates of TBx + standard SBx and TBx + perilesional SBx using a more strict definition of csPCa (ISUP GG ≥3). ciPCa clinically insignificant prostate cancer, csPCa clinically significant prostate cancer, TBx targeted biopsies, SBx systematic biopsies.

| Supplementary Table 3. Cancer detection rates of TBx + Perilesional SBx and TBx + Standard SBx in reference to TBx | | | | | | | | |
| --- | --- | --- | --- | --- | --- | --- | --- | --- |
|  | | **TBx + Standard SBx** | | | **TBx + Perilesional SBx** | | |  |
|  |  | csPCa (GG≥2) | ciPCa (GG=1) | Benign | csPCa (GG≥2) | ciPCa (GG=1) | Benign | Total |
| **TBx** | csPCa (GG≥2) | 89 | 0 | 0 | 89 | 0 | 0 | 89 |
|  | ciPCa (GG=1) | *6* | 72 | 0 | *3* | 75 | 0 | 78 |
|  | Benign | 0 | *14* | 54 | 0 | *3* | 65 | 68 |
| **Total** | | 95 | 86 | 54 | 92 | 78 | 65 | 235 |

**Supplementary Table 3.** Cancer detection rates of TBx, TBx + standard SBx and TBx + perilesional SBx. ciPCa clinically insignificant prostate cancer, csPCa clinically significant prostate cancer, TBx targeted biopsies, SBx systematic biopsies, PI-RADS prostate imaging reporting and data system.

| Supplementary Table 4. Cancer detection rates of TBx + Perilesional SBx and TBx + Standard SBx by PI-RADS-category in reference to TBx | | | | | | | | | | | |
| --- | --- | --- | --- | --- | --- | --- | --- | --- | --- | --- | --- |
|  | | | | **TBx + Standard SBx** | | | **TBx + Perilesional SBx** | | |  |  |
|  |  |  |  | csPCa (GG≥2) | ciPCa (GG=1) | Benign | csPCa (GG≥2) | ciPCa (GG=1) | Benign | Total | |
| **PI-RADS 3** | **TBx** | | csPCa (GG≥2) | 3 | 0 | 0 | 3 | 0 | 0 | 3 | |
|  |  |  | ciPCa (GG=1) | *3* | 6 | 0 | *2* | 7 | 0 | 9 | |
|  |  |  | Benign | 0 | *3* | 18 | 0 | *0* | 21 | 21 | |
|  | **Total** | | | 6 | 9 | 18 | 5 | 7 | 21 | 33 | |
| **PI-RADS 4** | **TBx** | | csPCa (GG≥2) | 39 | 0 | 0 | 39 | 0 | 0 | 39 | |
|  |  |  | ciPCa (GG=1) | *1* | 43 | 0 | *0* | 44 | 0 | 44 | |
|  |  |  | Benign | 0 | *9* | 27 | 0 | *2* | 34 | 36 | |
|  | **Total** | | | 40 | 52 | 27 | 39 | 46 | 34 | 119 | |
| **PI-RADS 5** | **TBx** | csPCa (GG≥2) | | 47 | 0 | 0 | 47 | 0 | 0 | 47 | |
|  |  | ciPCa (GG=1) | | *2* | 23 | 0 | *1* | 24 | 0 | 25 | |
|  |  | Benign | | 0 | *2* | 9 | 0 | *1* | 10 | 11 | |
|  | **Total** | | | 49 | 25 | 9 | 48 | 25 | 10 | 83 | |

**Supplementary Table 4.** Cancer detection rates of TBx, TBx + standard SBx and TBx + perilesional SBx by PI-RADS assessment category. ciPCa clinically insignificant prostate cancer, csPCa clinically significant prostate cancer, TBx targeted biopsies, SBx systematic biopsies, PI-RADS prostate imaging reporting and data system.
